# Copy number alterations detected by whole-exome and whole-genome sequencing of esophageal adenocarcinoma

**DOI:** 10.1186/s40246-015-0044-0

**Published:** 2015-09-15

**Authors:** Xiaoyu Wang, Xiaohong Li, Yichen Cheng, Xin Sun, Xibin Sun, Steve Self, Charles Kooperberg, James Y. Dai

**Affiliations:** Vaccine and Infectious Disease Division, Fred Hutchinson Cancer Research Center, Seattle, WA USA; Human Biology, Fred Hutchinson Cancer Research Center, Seattle, WA USA; Public Health Science Division, Fred Hutchinson Cancer Research Center, Seattle, WA USA; Institute of Occupational Health and Poison Control, Chinese Center for Disease Control and Prevention, Beijing, China; Henan Office for Cancer Research and Control, Henan Cancer Hospital, Zhengzhou, Henan China

## Abstract

**Background:**

Esophageal adenocarcinoma (EA) is among the leading causes of cancer mortality, especially in developed countries. A high level of somatic copy number alterations (CNAs) accumulates over the decades in the progression from Barrett’s esophagus, the precursor lesion, to EA. Accurate identification of somatic CNAs is essential to understand cancer development. Many studies have been conducted for the detection of CNA in EA using microarrays. Next-generation sequencing (NGS) technologies are believed to have advantages in sensitivity and accuracy to detect CNA, yet no NGS-based CNA detection in EA has been reported.

**Results:**

In this study, we analyzed whole-exome (WES) and whole-genome sequencing (WGS) data for detecting CNA from a published large-scale genomic study of EA. Two specific comparisons were conducted. First, the recurrent CNAs based on WGS and WES data from 145 EA samples were compared to those found in five previous microarray-based studies. We found that the majority of the previously identified regions were also detected in this study. Interestingly, some novel amplifications and deletions were discovered using the NGS data. In particular, *SKI* and *PRKCZ* detected in a deletion region are involved in transforming growth factor-β pathway, suggesting the potential utility of novel biomarkers for EA. Second, we compared CNAs detected in WGS and WES data from the same 15 EA samples. No large-scale CNA was identified statistically more frequently by WES or WGS, while more focal-scale CNAs were detected by WGS than by WES.

**Conclusions:**

Our results suggest that NGS can replace microarrays to detect CNA in EA. WGS is superior to WES in that it can offer finer resolution for the detection, though if the interest is on recurrent CNAs, WES can be preferable to WGS for its cost-effectiveness.

**Electronic supplementary material:**

The online version of this article (doi:10.1186/s40246-015-0044-0) contains supplementary material, which is available to authorized users.

## Background

Cancer arises from gradual accumulation of somatic genomic instability and alterations, which eventually lead to carcinogenesis and cancer progression [1, 2]. Copy number alterations (CNAs), one form of somatic genome alterations, refer to somatic changes in chromosome structure that result in gains or losses of copies of DNA segments. Detection of CNA is important to understand cancer development and identify key driver events [3, 4]. Microarray technologies have been widely used in CNA detection [5–7], including array comparative genomic hybridization (array CGH) and single nucleotide polymorphisms (SNP) microarrays. In array CGH, reference and test DNAs are fluorescence-labeled and hybridized to arrays, which are composed of bacterial artificial chromosome (BAC) clones, cDNA clones, or oligonucleotides. The signal ratio is used as an estimate of the copy number ratio. SNP microarrays are also based on hybridization, but a single sample is processed on each microarray and intensity ratios are formed by comparing the intensity of the sample under investigation to a collection of reference samples, or all other samples that are studied. Compared to array CGH, SNP arrays can have better resolution and produce B allele frequency so that loss of heterozygosity (LOH) can be detected [7]. Resolution of these arrays is typically greater than 1 kb, depending on the density, distribution, and response characteristics of their probes. More recently, next-generation sequencing (NGS) technologies offer single-nucleotide resolution and absolute counts of read numbers and therefore can provide more sensitive and accurate CNA results. Moreover, direct sequencing enables substantial increases in discoveries of smaller structural variation events [8, 9]. It is believed that, with its ever-decreasing cost, NGS will ultimately replace microarrays in copy number analyses [10].

In this paper, we conduct CNA analyses using published NGS data from [11], which contains 145 esophageal adenocarcinoma (EA) samples, as no CNA analyses were reported in the paper. The incidence of EA has strikingly increased over the past 30–40 years, and it is the seventh leading cause of cancer death among men in the USA [12]. Many studies of CNA detection in EA have been carried out using microarrays. Paulson et al. detected 19 most frequent CNAs in 15 EA patients using BAC array data [13]. Beroukhim et al. created the Tumorscape Copy Number Portal, where they collected more than 3000 copy number profiles from 26 cancer types using Affymetrix 250K StyI (Affymetrix, Santa Clara, CA) arrays [3]. They identified 33 recurrent CNAs (RCNAs), which appear in 44 EA samples more frequently than expected by chance. Dulak et al. detected 46 regions of significant recurrent events of gain and loss in 186 EA samples using 250K StyI arrays and SNP Array 6.0 arrays (Affymetrix) [14]. Zack et al. created the TCGA Copy Number Portal and identified RCNAs across multiple cancer types; they detected 88 RCNAS across 184 EA samples using Affymetrix SNP6 arrays [4, 15]. Frankel et al. detected 52 RCNAs in 54 EA samples using Illumina CytoSNP-12 arrays [16]. However, there has not been any published CNA detection study using NGS technologies. In this study, we plan to fill the gap by analyzing the NGS data from [11] and compare the result to the findings of the aforementioned papers.

Indeed, microarray-based CNA analyses are still a common approach to detect CNAs, possibly due to the following reasons: microarray technologies have been developed for a longer time and corresponding CNA detection methods were well established and accurate detection of CNA in NGS can be a challenging task due to the complexities of sequencing data processing [17]. To the best of our knowledge, only a few CNA studies have been conducted to compare the performance of microarrays and NGS side-by-side. Koboldt et al. detected CNAs on coding regions of five ovarian tumors using both a SNP array and two NGS platforms—whole-genome (WGS) and whole-exome sequencing (WES) [18]. They found the majority of CNA events were consistently detected by the three platforms. More CNAs were detected by the WGS platform than those by the array. In another study, the authors detected germline copy number variations (CNVs) in 16 breast cancer cell lines using both array CGH and WES [19]. Four WES-based CNV detection methods were compared, and the regions detected by the array were used to form the gold standard. They detected a greater number of focal-scale CNVs using the array. These studies were conducted on the individual sample level. In this study, we are interested to detect and compare regions frequently appearing among multiple samples between NGS data and previous findings derived from microarrays-based studies. The detected recurrent regions may contain real driver events that contribute to the cancer development.

Furthermore, there were 15 samples (patients) subjected to both WGS and WES in [11], providing a great opportunity to compare CNA detection by WES and WGS. Not much work has been conducted to address this question. Koboldt et al. found that a significant portion (79.53 %) of focal-scale CNAs detected by WES were also supported by WGS, and they recommended the use of WES-based approach, by which it is likely to detect more platform-specific focal copy number changes missed by WGS and microarray [18]. WES is an increasingly popular platform for studying tumor genomics because of itscost-effectiveness and the immediate interpretation of mutations in coding regions. It has been shown that WES data can be used to study CNA [19]. However, the uniformity of WES coverage is worse than that of WGS mostly due to exome capturing, and exons are not evenly placed within the genome so that it is difficult to detect CNAs over a long intergenic region using WES. On the other hand, if the interest is long CNA segments spanning over genes, it is not clear whether CNAs inferred by WES will lose a substantial amount of information when compared to WGS. It is quite possible that this comparison may depend on cancer site and the length of CNAs, since longer segment should be reliably detected by exome sequencing.

A number of bioinformatics and statistical methods have been developed for CNA detection using NGS data [17, 20–22]. These methods can be classified in several ways. Most methods were developed to detect CNAs on the individual sample level, and they usually detect CNAs based on read count ratios between a tumor sample and its matched normal sample. These methods can be further categorized according to the study design. Some commonly used ones are as follows. (a) CNVnator [23], RDXplorer [24], and ReadDepth [25] detect CNAs on a single tumor sample. (b) CNAseg [26], Segseq [27], ExomeCNV [28], HMMcopy [29], and VarScan2 [18] identify CNAs on matched tumor-normal samples. Control-FREEC [30, 31] can be categorized both into classes (a) and (b), as it can either work with tumor-normal pairs or with tumor-only samples. Depending on the NGS platforms, CNVnator, Segseq, RDXplorer, ReadDepth, and HMMcopy work for WGS data; ExomeCNV and VarScan work for WES data; and Control-FREEC can work for both types of the sequencing data. In addition to the above methods detecting CNA in individual samples, other methods have been developed to detect RCNAs from multiple samples. These methods take segments from all the individual samples as input and identify the (merged) segments which appear more frequently across the population than expected by chance. Only a few RCNA methods have been developed for NGS data, including JointSLM [32] and cn.MOPS [33]. They conduct copy number analyses based on read counts of segments of multiple tumor samples and usually are applied for CNV detection. On the other hand, many RCNA detection methods that were originally developed for microarray platforms [34] can also be adapted to work on NGS data. These methods include STAC [35], CMDS [36], and GISTIC2.0 [37].

In this study, Control-FREEC is selected to detect CNAs on the individual sample level using WGS and WES data from [11], and the results are compared between the two sequencing platforms. Control-FREEC is a flexible and powerful tool in that it performs multiple types of bias corrections considering GC-content, mappability, and matched normal sample, and it is among the most sensitive tools on both WGS and WES platforms [22]. GISTIC2.0, likely the most popular RCNA detection method, is chosen to detect RCNAs using both WGS and WES data. The identified RCNAs are then compared with those reported previously using microarrays. We compare our results with those from five previous studies, and four of which (all except [13]) used GISTIC2.0. By choosing GISTIC2.0, we hope to alleviate the concern that potential differences generated in the NGS data are due to different software and analytical methods being applied.

## Results

### RCNA analysis

**Fig. 1:**
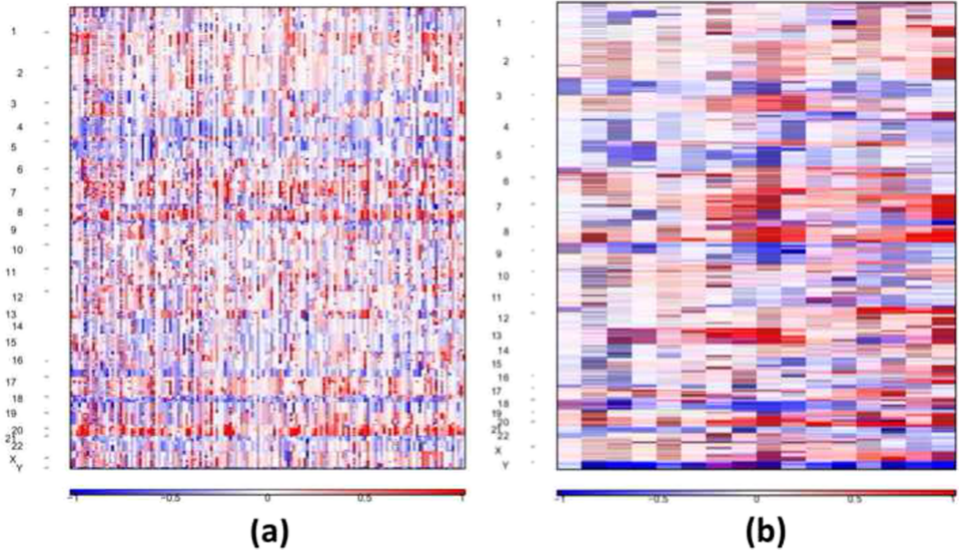
Segmented copy number ratio profiles in WES and WGS. The *x*-axis represents the samples. The *y*-axis represents the chromosomes. **a** WES data. **b** WGS data

**Fig. 2:**
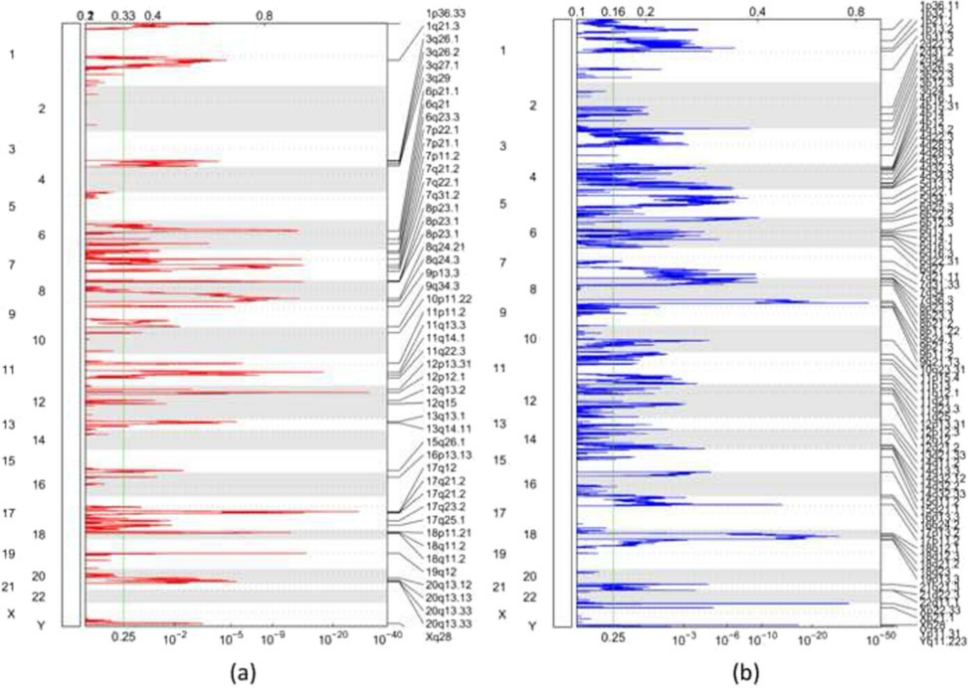
Genomic positions of RCNAs detected in 145 WES data. The *x*-axis represents the normalized amplification signals (*top*) and significance by *q* value (*bottom*). The *green line* indicates the significance cutoff at *q* = 0.25. **a** Amplification regions. **b** Deletion regions

**Fig. 3:**
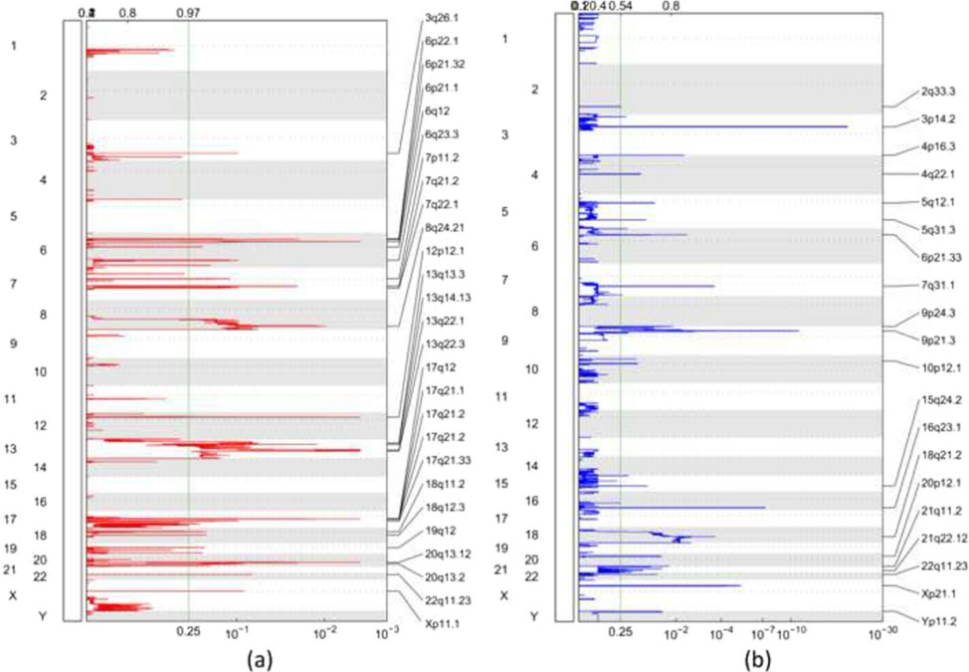
Genomic positions of RCNAs detected in 15 WGS data. The *x*-axis represents the normalized amplification signals (*top*) and significance by *q* value (*bottom*). The green line indicates the significance cutoff at *q* = 0.25. **a** Amplification regions. **b** Deletion regions

The estimated copy ratios of segments among 145 WES and 15 WGS data are shown in Fig. 1. We used GISTIC2.0 on the copy ratio profiles to perform a permutation-based significance analysis and identify significantly amplified/deleted regions. The recurrent amplification/deletion regions for WES data are shown in Fig. 2. The results of WGS data are shown in Fig. 3 accordingly. The threshold for the residual *q* value was set as 0.1, resulting in 41/16 amplifications and 67/19 deletions in WES/WGS data, respectively. We further combined the results from WES and WGS, and resulted in 47 amplification and 74 deletion events.

These newly identified genomic regions were verified with all the five microarray-based studies (Tables 1 and 2). It was found that the majority of the regions (68 % of deletions and 74 % of amplifications) detected in our study were also identified in those previous studies. Known cancer genes within these regions were identified according to the Cancer Gene Census [38], and the results are shown in the supplementary document (Additional file 1: Tables S1 and S2). Among all these detected regions, 13 amplification events were not reported in any of the previous studies; four of them (1p36.33, 12p13.31, 18p11.21, 8q24.3) had a residual *q* value less than 0.01. Twenty-nine deletion events were not identified previously, and ten of them (Xp22.33, 3p26.3, 6q22.31, 14q32.2, 1p21.1, 3p12.3, 6q12, Yq12, 6p12.3, 14p11.2) had a residual *q* value less than 0.01. We also examined the regions identified from the five previous studies to see whether they were also identified using the NGS data. We extracted the amplification regions (from Additional file 1: Table S2-C) and deletion regions (from Additional file 1: Table S4-B) in [14], for example. We checked if these regions were detected using the sequencing data and listed the *q* value for each region in Table 3. The genomic location for each region was converted from hg18 to hg19 using the University of California, Santa Cruz (UCSC) liftOver tool. The majority of those regions overlapped with our results, except for four amplifications and four deletions. The comparisons with other four studies are listed in the supplementary document (Additional file 1: Tables S3–S6), from which it can be seen that 58 % of regions in [16], 95 % of regions in [13], 64 % of regions in [3], and 57 % of regions in [4] were detected in our study. From these comparisons, we observed that the majority of regions in previous microarray studies were detected using NGS data.

**Table 1:** Amplification RCNAs detected by 145 WES data and 15 WGS data

Cytoband	Peak boundary (Mb)	Width (Mb)	Platform	Residual *q* value	D	F	P	B	Z
1p36.33	chr1:0.99-3.16	2.174	WES	1.16E−04					
1q21.3	chr1:149.94-156.69	6.751	WES	2.14E−05	Y				
3q26.1	chr3:164.71-164.76	0.047	WES and WGS	9.75E−02					
3q26.2	chr3:169.43-170.59	1.158	WES	4.18E−02	Y	Y			Y
6p21.32	chr6:32.56-32.58	0.016	WGS	2.35E−02					
6p21.1	chr6:42.79-43.97	1.178	WES and WGS	2.00E−13	Y	Y			Y
6q23.3	chr6:135.29-135.71	0.421	WES and WGS	4.98E−04	Y				Y
7p22.1	chr7:4.29-6.89	2.609	WES	6.42E−02			Y		
7p11.2	chr7:55.00-55.46	0.455	WES	5.48E−14	Y	Y	Y	Y	Y
7q21.2	chr7:91.98-92.76	0.779	WES and WGS	5.07E−12	Y	Y	Y		Y
7q22.1	chr7:98.46-100.674	2.217	WES	7.81E−07	Y		Y	Y	
7q31.2	chr7:115.61-117.83	2.211	WES	1.71E−02	Y				
8p23.1	chr8:6.97-7.16	0.182	WES	5.08E−02	Y	Y			Y
8p23.1	chr8:7.37-7.63	0.263	WES	1.79E−03	Y	Y			Y
8p23.1	chr8:11.28-11.68	0.402	WES	4.25E−13	Y	Y			Y
8q24.21	chr8:126.45-129.02	2.572	WES and WGS	1.79E−10	Y	Y	Y	Y	Y
8q24.3	chr8:141.9-146.36	4.464	WES and WGS	1.19E−03					
9p13.3	chr9:35.4-35.97	0.571	WES	5.60E−06	Y				Y
9q33.3	chr9:124.98-141.21	16.234	WES	6.22E−03					Y
10p11.22	chr10:31.61-33.62	2.015	WES	9.75E−02					
11p11.2	chr11:46.1-47.18	1.076	WES	1.30E−06					Y
11q13.3	chr11:68.86-69.63	0.775	WES	5.25E−16	Y	Y		Y	Y
11q14.1	chr11:77.73-77.88	0.157	WES	8.70E−05					Y
12p13.31	chr12:9.63-9.72	0.082	WES	7.09E−03					
12p12.1	chr12:25.34-25.67	0.328	WES and WGS	2.63E−32	Y	Y		Y	Y
12q13.3	chr12:56.14-57.32	1.181	WES	8.56E−02					
12q15	chr12:67.07-70.19	3.116	WES	3.88E−02	Y	Y		Y	Y
13q13.2	chr13:33.28-35.25	1.972	WES	8.76E−04	Y				
13q14.11	chr13:39.36-43.16	3.798	WES	1.99E−02	Y				
13q14.13	chr13:44.73-46.64	1.906	WGS	7.11E−02					
13q22.1	chr13:72.13-78.67	6.54	WGS	5.39E−02		Y		Y	Y
15q26.1	chr15:90.03-91.79	1.765	WES	4.45E−03			Y		Y
16p13.13	chr16:11.37-12.01	0.64	WES	3.88E−02					
17q12	chr17:37.83-37.9	0.072	WES	1.04E−24	Y	Y	Y	Y	Y
17q21.2	chr17:38.82-39.02	0.2	WES and WGS	4.35E−02	Y				
17q21.2	chr17:39.85-39.99	0.146	WES and WGS	1.90E−08	Y				
17q21.33	chr17:48.68-49.12	0.442	WGS	9.71E−02					
17q25.3	chr17:68.13-81.20	13.067	WES	5.04E−02					
18p11.21	chr18:12.25-13.44	1.184	WES	9.23E−03					
18q11.2	chr18:19.75-20.52	0.766	WES	5.94E−11	Y	Y	Y		Y
19q12	chr19:30.19-30.48	0.282	WES	6.69E−15	Y	Y	Y	Y	Y
20q13.12	chr20:42.98-43.56	0.584	WES and WGS	8.36E−02			Y		
20q13.2	chr20:47.90-52.77	4.877	WES	1.00E−02		Y	Y		
20q13.33	chr20:58.42-58.51	0.099	WES	1.75E−03			Y		
22q11.23	chr22:24.39-24.41	0.016	WGS	7.11E−02					
Xp11.1	chrX:58.52-58.53	0.014	WGS	8.53E−02		Y			
Xq28	chrX:152.11-153.91	1.793	WES	5.53E−04					Y

A region may span multiple cytobands, in which case the longest one was listed. The regions were verified by checking if they were identified in any of the five previous microarray-based studies

*D* Dulak et al. 2012 [14], *F* Frankel et al. 2014 [16], *P* Paulson et al. 2009 [13], *B* Beroukhim et al. 2010 [3], *Z* Zack et al. 2013 [4], *Y* indicates a region was identified in a study

**Table 2:** Deletion RCNAs detected by 145 WES data and 15 WGS data

Cytoband	Boundary (Mb)	Width (Mb)	Platform	Residual *q* value	D	F	P	B	Z
1p36.11	chr1:19.53-31.73	12.21	WES	9.12E−04	Y			Y	Y
1p31.1	chr1:45.53-100.32	54.79	WES	5.89E−02					
1p21.1	chr1:104.12-107.60	3.48	WES	2.86E−04					
1p13.2	chr1:115.32-115.58	0.26	WES	5.65E−04					Y
1q31.3	chr1:186.41-200.18	13.77	WES	1.16E−02					
2q22.1	chr2:136.87-149.40	12.53	WES	8.62E−02				Y	Y
2q32.1	chr2:179.23-190.43	11.20	WES	6.27E−02					
3p26.3	chr3:0.00-2.61	2.61	WES	3.97E−08					
3p24.3	chr3:12.79-69.03	56.24	WES and WGS	3.19E−02	Y	Y		Y	Y
3p12.3	chr3:75.71-88.10	12.39	WES	2.29E−03					
4p16.1	chr4:0.00-15.97	15.97	WES and WGS	2.81E−02					
4p15.31	chr4:17.84-24.53	6.69	WES	9.06E−03					Y
4p12	chr4:42.15-47.45	5.30	WES	5.30E−02					
4q13.2	chr4:69.34-71.25	1.91	WES	2.49E−02					
4q22.1	chr4:90.88-93.23	2.35	WGS	9.73E−02	Y	Y			Y
4q28.3	chr4:129.78-139.98	10.20	WES	3.52E−02					
4q32.1	chr4:154.56-159.59	5.03	WES	9.27E−02		Y			
4q32.3	chr4:164.44-165.88	1.44	WES	1.84E−02					
4q34.3	chr4:174.30-191.15	16.86	WES	2.50E−03	Y			Y	Y
5q12.1	chr5:58.15-59.79	1.64	WGS	4.42E−02	Y	Y	Y	Y	Y
5q13.1	chr5:66.46-68.46	2.00	WES	3.52E−06			Y		
5q14.3	chr5:79.47-130.52	51.04	WES	1.41E−03			Y		
6p25.3	chr6:0.00-2.62	2.62	WES	3.97E−07	Y	Y			Y
6p12.3	chr6:49.82-50.79	0.97	WES	8.30E−03					
6p22.2	chr6:24.98-25.73	0.75	WES	1.97E−02					
6p21.33	chr6:31.17-31.32	0.15	WGS	3.97E−03					
6q12	chr6:64.42-71.14	6.72	WES	3.62E−03					
6q16.1	chr6:90.58-97.25	6.67	WES	2.08E−02					Y
6q16.3	chr6:100.06-105.41	5.35	WES	4.82E−02					Y
6q22.31	chr6:123.37-124.60	1.23	WES	1.21E−04					
6q27	chr6:151.79-171.12	19.33	WES	2.27E−02	Y			Y	Y
7q31.1	chr7:105.14-128.47	23.33	WES and WGS	8.30E−03	Y			Y	Y
7q34	chr7:141.64-141.95	0.31	WES	2.45E−06	Y			Y	
8p23.2	chr8:0.00-6.26	6.27	WES	9.89E−05	Y		Y	Y	Y
8p23.1	chr8:7.83-10.39	2.55	WES	2.59E−02					
8p21.2	chr8:23.42-24.77	1.35	WES	1.73E−05			Y		
8p11.22	chr8:38.85-39.78	0.92	WES	5.30E−02				Y	
9p23	chr9:6.64-15.17	8.53	WES	1.10E−06	Y	Y	Y		Y
9p21.3	chr9:21.86-23.69	1.83	WES and WGS	1.35E−34	Y	Y	Y	Y	Y
9q12	chr9:43.13-66.51	23.38	WES	3.52E−02					
9q31.1	chr9:70.49-123.15	52.66	WES	2.49E−02					
10q23.31	chr10:89.55-94.21	4.67	WES	2.63E−05					Y
11p15.4	chr11:0.00-8.94	8.94	WES	1.05E−02					Y
11p11.12	chr11:49.00-57.07	8.07	WES	8.36E−02					
11q14.1	chr11:77.96-111.96	33.99	WES	6.27E−02	Y				
11q25	chr11:126.13-135.01	8.88	WES	4.23E−03	Y			Y	Y
12p13.31	chr12:9.47-9.75	0.29	WES	4.82E−02					
12q12	chr12:33.56-48.13	14.57	WES	5.89E−02					
12q21.31	chr12:70.76-93.77	23.01	WES	4.23E−03		Y			
13q31.1	chr13:61.10-95.23	34.12	WES	2.38E−02					
14p11.2	chr14:0.00-20.48	20.48	WES	8.69E−03					
14q13.3	chr14:36.79-37.64	0.86	WES	5.30E−02					Y
14q32.13	chr14:94.16-96.85	2.69	WES	1.39E−02					
14q32.2	chr14:97.03-107.35	10.32	WES	2.37E−04					
15q11.2	chr15:20.78-22.69	1.91	WES	2.59E−02		Y	Y		Y
15q24.2	chr15:74.01-77.71	3.71	WGS	6.84E−02					
16p13.3	chr16:0.00-4.90	4.90	WES	2.10E−05					Y
16q21	chr16:29.48-90.35	60.88	WES and WGS	7.29E−02	Y	Y	Y		Y
17p12	chr17:0.00-18.02	18.02	WES	3.43E−03			Y		
17p11.2	chr17:18.42-18.54	0.12	WES	2.20E−13			Y		Y
18q12.1	chr18:24.60-28.65	4.04	WES and WGS	6.80E−03			Y		Y
18q12.3	chr18:35.15-42.28	7.14	WES and WGS	5.53E−07			Y		Y
18q21.2	chr18:48.59-50.28	1.69	WES and WGS	2.87E−13	Y	Y	Y		Y
18q23	chr18:67.87-78.08	10.21	WES and WGS	8.67E−05			Y	Y	Y
19p13.3	chr19:0.00-10.66	10.65	WES	6.47E−04					Y
20p12.1	chr20:13.97-16.04	2.06	WGS	2.93E−02	Y	Y			Y
21p11.2	chr21:0.00-15.32	15.32	WGS	1.77E−02	Y				
21q21.1	chr21:19.63-27.01	7.38	WES	4.49E−05			Y		Y
21q22.3	chr21:47.86-48.13	0.27	WES and WGS	2.64E−04			Y		
22q11.23	chr22:24.33-24.37	0.05	WGS	3.83E−02			Y		
Xp22.33	chrX:0.00-2.67	2.67	WES	8.13E−33					
Xp21.1	chrX:30.87-32.66	1.79	WES and WGS	2.99E−04		Y			Y
Xq28	chrX:154.75-155.27	0.52	WES	2.55E−17		Y			
Yq12	chrY:20.89-59.22	38.33	WES and WGS	4.08E−03					

A region may span multiple cytobands, in which case the longest one was listed. The regions were verified by checking if they were identified in any of the five previous microarray-based studies

*D* Dulak et al. 2012 [14], *F* Frankel et al. 2014 [16], *P* Paulson et al. 2009 [13], *B* Beroukhim et al. 2010 [3], *Z* Zack et al. 2013 [4], *O* our study, *Y* indicates a region was identified in a study

**Table 3:** Comparison of results of Dulak et al. [14] to our results

Cytoband	Boundary (Mb)	Width (Mb)	Our study	Residual *q* value	Type
12p12.1	chr12:25.34-25.45	0.11	Y	2.63E−32	amp
18q11.2	chr18:19.70-19.91	0.21	Y	5.94E−11	amp
8p23.1	chr8:11.37-11.67	0.30	Y	4.25E−13	amp
19q12	chr19:30.25-30.41	0.16	Y	6.69E−15	amp
7q21.2	chr7:92.48-92.66	0.18	Y	5.07E−12	amp
11q13.3	chr11:69.26-69.81	0.55	Y	5.25E−16	amp
17q12	chr17:37.72-38.02	0.30	Y	1.04E−24	amp
17q21.2	chr17:39.77-39.96	0.19	Y	1.90E−08	amp
7p11.2	chr7:54.95-55.43	0.48	Y	5.48E−14	amp
8q24.21	chr8:128.40-128.84	0.44	Y	1.79E−10	amp
6p21.1	chr6:43.21-43.35	0.14	Y	2.00E−13	amp
9p13.3	chr9:35.48-35.94	0.46	Y	5.60E−06	amp
13q13.1	chr13:33.38-34.43	1.05	Y	8.76E−04	amp
7q22.1	chr7:99.29-100.00	0.71	Y	7.81E−07	amp
7q31.2	chr7:116.13-116.63	0.50	Y	1.71E−02	amp
12q15	chr12:67.27-70.21	2.94	Y	3.88E−02	amp
6q23.3	chr6:135.28-135.83	0.55	Y	4.98E−04	amp
10q22.2	chr10:75.33-76.13	0.80			amp
1q21.3	chr1:147.76-154.10	6.34	Y	2.14E−05	amp
10q26.13	chr10:122.76-123.92	1.16			amp
3q26.2	chr3:168.72-172.28	3.56	Y	4.18E−02	amp
18q11.2	chr18:23.45-24.21	0.76	Y	5.94E−11	amp
13q14.11	chr13:41.37-41.93	0.56	Y	1.99E−02	amp
11p14.1	chr11:27.08-27.61	0.53			amp
7q34^a^	chr7:141.92-142.26	0.34			amp
3p14.2	chr3:58.98-61.54	2.56	Y	3.19E−02	del
16q23.1	chr16:78.13-79.65	1.52	Y	7.29E−02	del
9p21.3	chr9:21.86-22.02	0.16	Y	1.35E−34	del
5q12.1	chr5:58.26-59.79	1.53	Y	4.42E−02	del
6p25.3	chr6:1.60-2.63	1.03	Y	3.97E−07	del
20p12.1	chr20:14.26-16.04	1.78	Y	2.93E−02	del
4q22.1	chr4:91.15-93.27	2.12	Y	9.73E−02	del
18q21.2	chr18:48.52-48.72	0.20	Y	2.87E−13	del
21q22.12	chr21:36.11-36.43	0.32			del
9p23	chr9:7.79-12.72	4.93	Y	1.10E−06	del
6q26	chr6:161.69-163.21	1.52	Y	2.27E−02	del
2q33.3	chr2:204.82-206.56	1.74			del
1q44	chr1:245.85-246.71	0.86			del
8p23.3	chr8:1.01-1.46	0.45	Y	9.89E−05	del
7q33	chr7:123.66-142.53	18.87	Y	8.30E−03	del
7q36.1	chr7:148.11-159.13	11.02			del
1p36.11	chr1:25.77-31.25	5.48	Y	9.12E−04	del
4q34.3	chr4:178.82-185.31	6.49	Y	2.50E−03	del
11q22.3	chr11:105.95-112.83	6.88	Y	6.27E−02	del
11q25	chr11:121.03-134.94	13.91	Y	4.23E−03	del
21p11.2^a^	chr21:1.00-16.26	15.26	Y	1.77E−02	del

Regions detected in Dulak et al., 2012 [14] were verified in our study

^a^In cytoband indicates that the coordinate is based on hg18

To generate a consensus list of regions, we investigated all the genomic regions in terms of cytobands across all the results from the six studies including ours and listed the regions appearing in at least three of them. The results are shown in Tables 4 and 5. Only two amplifications and six deletions were not found in our study, and our result is the one that is most consistent with the consensus regions, which suggests that NGS may be a more powerful approach for detecting RCNAs.

**Table 4:** Consensus amplification RCNAs in 6 studies

Cytoband	Boundary (Mb)	D	F	P	B	Z	O
3q26.2	chr3:167.60-170.90	Y	Y			Y	Y
6p21.1	chr6:40.50-46.20	Y	Y			Y	Y
6q23.3	chr6:135.20-139.00	Y				Y	Y
7p11.2	chr7:54.00-58.00	Y	Y	Y	Y	Y	Y
7q21.2	chr7:91.10-92.80	Y	Y	Y		Y	Y
7q21.3	chr7:92.80-98.00		Y	Y	Y		
7q22.1	chr7:98.00-103.80	Y		Y	Y		Y
8p23.1	chr8:6.20-12.70	Y	Y			Y	Y
8q24.13	chr8:122.50-127.30			Y	Y		Y
8q24.21	chr8:127.30-131.50	Y	Y	Y	Y	Y	Y
9p13.3	chr9:33.20-36.30	Y				Y	Y
11q13.3	chr11:68.40-70.40	Y	Y		Y	Y	Y
12p12.1	chr12:21.30-26.50	Y	Y		Y	Y	Y
12q14.3	chr12:65.10-67.70	Y			Y		Y
12q15	chr12:67.70-71.50	Y	Y		Y	Y	Y
13q22.1	chr13:73.30-75.40		Y		Y	Y	Y
15q26.1	chr15:89.10-94.30			Y		Y	Y
15q26.2	chr15:94.30-98.50		Y	Y		Y	
17q12	chr17:31.80-38.10	Y	Y	Y	Y	Y	Y
18q 11.2	chr18:19.00-25.00	Y	Y	Y		Y	Y
19q12	chr19:28.60-32.40	Y	Y	Y	Y	Y	Y
20q13.2	chr20:49.80-55.00		Y	Y			Y

These regions are those that appear in at least three studies

*D* Dulak et al. 2012 [14], *F* Frankel et al. 2014 [16], *P* Paulson et al. 2009 [13], *B* Beroukhim et al. 2010 [3], *Z* Zack et al. 2013 [4], *O* our study, *Y* indicates a region was identified in a study

**Table 5:** Consensus deletion RCNAs in six studies

Cytoband	Boundary (Mb)	D	F	P	B	Z	O
1p36.11	chr1:23.90-28.00	Y			Y	Y	Y
1p35.3	chr1:28.00-30.20	Y			Y		Y
1p35.2	chr1:30.20-32.40	Y			Y		Y
1q44	chr1:243.70-249.25	Y	Y			Y	
2q22.1	chr2:136.80-142.20				Y	Y	Y
2q22.2	chr2:142.20-144.10				Y	Y	Y
2q33.3	chr2:204.90-209.00	Y			Y	Y	
3p14.2	chr3:58.60-63.70	Y	Y		Y	Y	Y
4q22.1	chr4:88.00-93.70	Y	Y			Y	Y
4q34.1	chr4:171.90-176.30				Y	Y	Y
4q34.2	chr4:176.30-177.50				Y	Y	Y
4q34.3	chr4:177.50-183.20	Y			Y	Y	Y
4q35.1	chr4:183.20-187.10	Y			Y	Y	Y
5q11.2	chr5:50.70-58.90	Y	Y	Y	Y	Y	Y
5q12.1	chr5:58.90-62.90	Y	Y	Y	Y	Y	Y
6p25.3	chr6:0.00-2.30	Y	Y			Y	Y
6p25.2	chr6:2.30-4.20	Y	Y				Y
6q26	chr6:161.00-164.50	Y			Y	Y	Y
7q31.1	chr7:107.40-114.60				Y	Y	Y
7q31.32	chr7:121.10-123.80	Y			Y		Y
7q31.33	chr7:123.80-127.10	Y			Y		Y
7q32.1	chr7:127.10-129.20	Y			Y		Y
7q34	chr7:138.20-143.10	Y			Y		Y
7q36.3	chr7:155.10-159.14	Y			Y	Y	
8p23.3	chr8:0.00-2.20	Y		Y	Y	Y	Y
8p23.2	chr8:2.20-6.20				Y	Y	Y
8p23.1	chr8:6.20-12.70				Y	Y	Y
9p24.1	chr9:4.60-9.00	Y	Y	Y		Y	Y
9p23	chr9:9.00-14.20	Y	Y	Y		Y	Y
9p21.3	chr9:19.90-25.60	Y	Y	Y	Y	Y	Y
11q24.2	chr11:123.90-127.80	Y			Y		Y
11q24.3	chr11:127.80-130.80	Y			Y	Y	Y
11q25	chr11:130.80-135.01	Y			Y	Y	Y
16q23.1	chr16:74.10-79.20	Y	Y	Y		Y	Y
16q23.2	chr16:79.20-81.70	Y	Y			Y	Y
17p11.2	chr17:16.00-22.20			Y		Y	Y
18q12.2	chr18:32.70-37.20			Y		Y	Y
18q12.3	chr18:37.20-43.50			Y		Y	Y
18q21.2	chr18:48.20-53.80	Y	Y	Y		Y	Y
18q21.33	chr18:59.00-61.60			Y	Y	Y	
18q22.1	chr18:61.60-66.80			Y	Y	Y	
18q22.2	chr18:66.80-68.70			Y	Y	Y	Y
18q22.3	chr18:68.70-73.10			Y	Y	Y	Y
18q23	chr18:73.10-78.077			Y	Y	Y	Y
20p12.1	chr20:12.10-17.90	Y	Y			Y	Y
21q11.2	chr21:14.30-16.40	Y		Y		Y	Y
21q21.1	chr21:16.40-24.00			Y		Y	Y
21q21.2	chr21:24.00-26.80			Y		Y	Y
21q22.12	chr21:35.80-37.80	Y	Y	Y		Y	
Xp21.2	chrX:29.30-31.50		Y			Y	Y
Xp21.1	chrX:31.50-37.60		Y			Y	Y

These regions are those that appear in at least three studies

### Comparison of CNAs on WGS and WES

We detected CNAs in 15 normal-tumor sample pairs based on both WGS data and WES data using Control-FREEC and compared the results from the two platforms. The comparisons were made on different lengths of segments, including large-scale and focal-scale, where large-scale CNAs refer to those spanning more than 25 % of a chromosome arm and focal-scale CNAs refer to those shorter than 25 % of a chromosome arm. The size span of large-scale CNAs is [18.32 161.22] Mb, with a standard deviation of 37.39 Mb. The size span of small-scale CNAs is [0.001 50.65] Mb, with a standard deviation of 2.50 Mb. More than 83 % of focal-scale CNAs are shorter than 1 Mb. For each detected CNA, we used Kolmogorov-Smirnov (KS) test to assess the possibility that it was generated just by chance; furthermore, we searched the WGS and WES data of each sample to see if it contained an event that overlapped the detected CNA with at least 10 % of bases, i.e., we counted how many times it appeared in WGS data and WES data. We then applied Fisher’s exact test to compare the detection frequency of each CNA by the two platforms.

The results of large-scale CNAs are shown in Table 6. Totally, 19 regions were detected from the 15 EA samples. We then counted how many times these CNAs were detected by WGS and WES and found none of them was more frequently detected by one platform than the other. In addition, we used KS test and found the false-positive detection rate of each identified CNA was 0.

**Table 6:** Large-scale CNAs detected in WGS and WES

Boundary (Mb)	Width (Mb)	Type	WGS count	WES count	*p* value
chrX: 2.70-44.00	41	Gain	0	1	1.00
chr12:58.34-110.00	52.05	Gain	1	3	0.60
chr8:87.08-126.00	39.3	Gain	0	1	1.00
chr14:37.15-65.00	27.5	Gain	0	1	1.00
chr20:36.79-55.00	18.32	Gain	0	1	1.00
chr4:20.88-69.00	48.46	Gain	0	1	1.00
chrX:46.95-139.00	91.93	Loss	1	4	0.33
chr8:0.12-86.00	86.27	Loss	1	2	1.00
chr13:35.76-115.00	79.35	Loss	2	5	0.39
chr4:75.26-178.00	103.1	Loss	1	3	0.60
chr5:19.47-181.00	161.22	Loss	2	4	0.65
chrY:10.01-29.00	18.71	Loss	0	5	0.04
chr17:0.00-21.00	21.19	Loss	2	1	1.00
chr18:19.84-78.00	58.18	Loss	5	4	1.00
chr15:32.02-60.00	28.39	Loss	1	0	1.00
chr19:0.25-25.00	24.26	Loss	3	0	0.22
chr19:36.81-55.00	18.42	Loss	1	0	1.00
chr7:93.74-152.00	58.36	Loss	2	1	1.00
chr21:15.00-45.00	29.63	Loss	1	1	1.00

*p* value is used to assess if the detected CNA is more frequently identified by WGS or WES

The results of focal-scale CNAs are shown in Table 7. WGS identified 21,197 focal-scale CNAs from the 15 samples; among them, 3675 were statistically more frequently detected by WGS than by WES. WES identified 4371 focal-scale CNAs, and 144 of them were identified more frequently by the platform. We checked the false-positive detection rates of the detected CNAs using the KS test and found 19,694/3655 CNAs on WGS/WES with *p* values < 0.05; these CNAs are less likely to be spurious discoveries, and we only worked on these CNAs afterwards. Among them, about 18 % of CNAs detected by WGS were statistically more frequently identified by WGS than by WES, while only about 3 % of CNAs detected by WES were more frequently identified on the platform. We further investigated if the false-positive detection rates of small CNAs (<200 k) detected on the two platforms were different using one-tailed *t* test, which resulted in a *p* value of 2.2E−16 (with means 0.004 vs. 0.009), and it indicates that the false-positive detection rate of those small CNAs is significantly smaller using WGS. One possible explanation is that WGS does not contain the exome-capturing process as in WES, and the local variation/bias of sequence read coverage is smaller [39]. Compared to WGS, WES does not cover intron regions, and it only covers 2.76 % of the whole genome. So finally, we investigated the effect of non-coverage to CNA detection and dealt with small CNAs that only reside in intron regions. As the result, no CNAs detected by WES spanned only on introns, and more than 7000 of such CNAs were identified by WGS, but only 22 % of these intron CNAs were statistically more frequently detected by WGS.

**Table 7:** Focal-scale CNAs detected in WGS and WES

	WGS	WES
All CNAs	21,197/3675	4371/144
Filtered CNAs	19,694/3456	3655/121
Small CNAs (<200 k)	11,480/2175	1201/36
Small and intron CNAs	7452/1603	0/0

A number before/indicates how many CNAs detected in the specific platform; a number after/indicates how many CNAs are more frequently detected in the platform. Filtered CNAs represent detected CNAs with *p*-value < 0.05. Small and Intron CNAs are CNAs with width < 200 k and only cover introns

## Discussion

In this study, we detected RCNAs using NGS data from 145 EA samples and compared them with those from the five microarray studies. We found that the majority of the regions detected by microarrays overlapped the regions identified by NGS and vise versa. Furthermore, based on all these six studies, we identified 22/51 consensus amplification/deletion regions, and our result was found to be the one that is most concordant with the consensus events. From the above observations, we suggest that NGS can replace microarrays to detect RCNAs in EA.

However, discrepancy generally exists when comparing each specific region from all the studies. Even for the largest detected events, they are not consistent across the platforms and across the different microarray studies. The largest recurrent deletions detected by microarrays are not consistent. Two of them [3, 14] identified the largest recurrent deletions on chr7:123.66-142.52 (Mb), which corresponds to chr7:105.14-128.47 (Mb) detected both by WGS and WES in our study. The largest deletion detected by WGS and WES is on Chr16:29.48-90.35 (Mb), while only part of the region—chr16:78.13–79.65 (Mb) (in [4, 14, 16]) and chr16:31.93–33.39 (Mb) (in [16])—were detected in the microarray studies. Part of these discrepancies may just be caused by different technologies used in these platforms, such as different hybridization and scanning methods applied in these microarray studies, target-enrichment strategies applied in WES, and bias due to the effect of GC-content and uneven mappability across genome in NGS. Although our study indicates a significant overlap between RCNAs detected using microarray data and NGS data, it is still a challenge to rigorously compare these RCNA calling methods. To further compare these approaches, a well-controlled study design such as a spike-in experiment should be applied in the future.

GISTIC analysis is often used to identify driver genes that contribute to cancer development. In this study, we found several potential driver genes in the detected regions that were reported in previous studies, and the results are listed in Table 8. We detected oncogenes such as *EGFR*, *ERBB2*, *GATA6*, *KRAS*, *MYC*, and tumor suppressor genes such as *APC*, *ARID1A*, *ATM*, *CDKN2A*, *CDKN2B*, *CDK6*, *MCL1*, *MET*, *MYB*, *PDE4D*, *PRCKI*, and *PTPRD*. Those were also identified in the various previous microarray studies. In another study [11], the authors identified 26 significantly mutated genes based on the 145 WES data used in our study. Among them, ten genes such as *TP53*, *CDKN2A*, *EYS*, *ARID1A*, *TLR4*, *ARID2*, *SYNE1*, *C6orf118*, *ACTL7B*, and *SCN10A* were also identified in our study, and three of the rest (*SMAD4*, *TLL1*, and *SMARCA4*) are located within 1 Mb of the detected regions of this study. It is worth to point out that some of the potential driver genes such as *ERBB2* and *TP53* were reported as implicated in the progression of esophageal Barrett to EA [13]. However, CNA regions are usually large and contain many genes. It is difficult to distinguish driver genes from passengers by just studying copy numbers [40]. Although more common driver genes were detected in this study than those found in [16], the discrepancy still implies the need of an integrated approach to identify driver genes of EA, which can consider CNA, mutation, gene expression, and methylation altogether.

**Table 8:** Potential driver genes reported in previous studies and corresponding RCNAs detected in this study

Genes	Cytoband	Boundary (Mb)
ARID1A	1p36.11	chr1:19.53-31.73
SKI.PRKCZ	1p36.33	chr1:0.99-3.16
MCL1	1q21.3	chr1:149.94-156.69
SCN10A	3p24.3	chr3:12.79-69.03
PRCKI	3q26.2	chr3:169.43-170.59
PDE4D	5q12.1	chr5:58.15-59.79
APC	5q14.3	chr5:79.47-130.52
EYS	6q12	chr6:64.42-71.14
MYB	6q23.3	chr6:135.29-135.71
C6orf118, SYNE1	6q27	chr6:151.79-171.12
EGFR	7p11.2	chr7:55.00-55.46
CDK6	7q21.2	chr7:91.98-92.76
MET	7q31.2	chr7:115.61-117.83
MYC	8q24.21	chr8:126.45-129.02
CDKN2A, CDKN2B	9p21.3	chr9:21.86-23.69
PTPRD	9p23	chr9:6.64-15.17
TLR4	9q31.1	chr9:70.49-123.15
ATM	11q14.1	chr11:77.96-111.96
KRAS	12p12.1	chr12:25.34-25.67
ARID2	12q12	chr12:33.56-48.13
GATA6	18q11.2	chr18:19.75-20.52
DMD	Xp21.1	chrX:30.87-32.66

In addition to the common regions, we found some novel ones, including four amplification regions and ten deletion regions with statistically high frequency of appearance in the population. These regions may provide more clues to understand the cancer genomics of EA. In particular, *SKI* and *PRKCZ* in 1p36.33 have been reported to contribute to the loss function of *TGFBR2* and *SMAD4* in cancer [41]. *TGFBR2* and *SMAD4* are involved in the transforming growth factor (TGF)-β pathway and were identified as driver genes in gastric cancer [42] and colorectal cancer [43]. The novel deletion event identified on Yq12 in our study, along with previously found deletion events on X chromosome (e.g., Xp21.1 and Xp21.2) may help to understand the greater incidence of EA in males over the past three decades. For example, the *DMD* gene in Xp21.1 was identified as a driver gene in gastric cancer [42], and our result suggests that it may also contribute to EA development.

The recurrently detected regions are likely to harbor “common mutations” that are of great interest in cancer studies. However, each tumor sample can contain private driver mutations for that individual patient’s tumor. To verify it, we compared the CNAs detected at individual sample level (Tables 6 and 7) with the recurrent events (Tables 1 and 2). We found only about 25.2 % of individual deletions overlapped identified deletion RCNAs. More extremely, only 10.2 % of amplifications detected at individual sample level overlapped those amplification RCNAs. Even for large-scale events, we found 88.0 % of individual deletions overlapped the recurrent deletion events, and only 35 % of individual amplifications overlapped the recurrent amplification events. The above observation implies that a considerable amount of driver mutations in a specific tumor sample is not located in the recurrent regions and personalized studies are required to identify these rare events.

In our study, the medians of spans of recurrent amplification/deletion events are 1.0/6.6 Mb for WES (and possibly WGS) and 0.2/2.1 Mb for those identified only from WGS (Tables 1 and 2). Also, we detected more individual small CNAs by WGS (Table 7). Compared to WES, WGS appears more powerful to detect small events, especially for those that mostly reside in non-coding regions. The limitation of this comparison is that only 15 WGS/WES samples were available. For future studies, a larger sample size should provide more precision to calibrate the performance of WES relative to WGS.

## Conclusions

In this study, we detected RCNAs in EA using the NGS data from [11] and compared the results with those from the previous microarray studies. The majority of the events detected in our study also were detected in those previous studies. Furthermore, novel regions and genes were found using NGS technologies. We also compared carefully WGS and WES in detecting CNA on an individual level. We found large-scale segments can be more consistently detected by both platforms, whereas WGS does detect more focal events. Importantly, the recurrent events on the population level appear to be successfully identified by WES. Given that the cost of WES is much less than that of WGS, and the mutations in WES is much more interpretable, our study suggests that WES may be the viable platform to detect recurrent copy number events in EA research.

## Methods

### Esophageal adenocarcinoma cancer data

The NGS data, including both WGS and WES data, were generated in [11] and stored in the database of Genotypes and Phenotypes (dbGaP) (study accession: phs000598.v1.p1). The dataset is comprised of 145 matched tumor-normal samples. Among them, 15 samples both have WGS and WES data, and the rest 130 samples have only WES data. The EA samples include those from the gastric-esophageal junction, not treated with chemotherapy or radiation before surgery. The tumor samples were examined by a board-certified pathologist and ensured that their carcinoma content >70 %. The samples were sequenced on multiple Illumina HiSeq flow cells to have the average target exome coverage of ~80× in WES data, and sequenced on the Illumina Genome Analyzer Iix or the Illumina HiSeq sequencer with an average of ~30× coverage depth in WGS data. The details of the sample collection, DNA extraction, and sequencing procedures can be found in [11].

The raw sequence data were extracted from dbGaP using the NCBI SRA Toolkit; the sequences were aligned to the NCBI build 37 (hg19) reference using BWA [44] and processed following GATK best practices. The base score re-calibrated bam files were used for CNA detection.

### CNA detection methods

Control-FREEC was applied in this study on both WGS and WES data. It divided the genome into small contiguous regions using sliding windows. The read count profiles in each region for normal and tumor samples were computed and normalized accounting for GC-content and mappability. The read count ratios of tumors to matched normal samples were calculated and used as the proxy of the copy number ratios. A LASSO-based algorithm was used to segment the data. LASSO is a widely used generalized linear regression method that involves penalizing the absolute size of its regression coefficients [45]. Using LASSO, a piecewise constant smoothed step profile was used to model the copy number ratios, and the positions with nonzero coefficients were considered as change points. For WES data, the window size was set to 500, and the step size was set to 250, which were recommended by the authors. For WGS data, those parameters were set as 2000 and 1000, respectively. Control-FREEC estimates the normal cell contamination in tumor samples by comparing the observed and predicted copy numbers. It uses the Kolmogorov-Smirnov test to assess the false-positive rate of each detected CNA. Control-FREEC can predict absolute copy numbers if the ploidy information is provided. We used ABSOLUTE [46] to estimate the ploidy of the 15 EA samples using WES data, and the results are listed in the supplement. In this study we classified the identified CNAs based on their status (amplification or deletion) instead of their absolute copy numbers. Control-FREEC ignored genomic regions with mappability less than 0.85 by default, and hence, we did not consider the effect of unmappable regions in this study.

GISTIC2.0 was used to identify regions with a statistically high frequency of copy number aberrations over background aberrations. It evaluated both the frequency and the significance to identify regions of interest. The G score measured both the frequency of aberrations, and the magnitude of the copy number changes (log ratio intensity) in each sample. Each location was scored separately for gains and losses. Then locations in each sample were permuted to simulate random aberrations. This random distribution was compared to the observed statistic to identify scores that are statistically significant. A false discovery rate (FDR) multiple testing correction was applied to calculate a *q*-bound significance score. Within each statistically significant region, a peak region was identified so that the region with a maximal G score and a minimal *q* value is most likely to contain affected genes. In addition to the *q* value, it also computed the residual *q* value, which measured the *q* value of a peak region after removing events that overlap with other more significant peak regions in the same chromosome. The 145 WES data were segmented using circular binary segmentation (CBS) algorithm [47] and combined to form the segmentation file, while the 15 WGS data were segmented using Control-FREEC as described above. The parameter settings were as follows: amplification threshold = 0.1, deletion threshold = 0.1, broad length cutoff = 0.98, remove X-chromosome = 0, and confidence level = 0.95.

Whenever possible, default parameters and recommended settings were used in the implementation of these tools.

## Additional file

Additional file 1:
**Supplementary tables.** **Table S1.** Cancer genes in detected amplification regions. **Table S2.** Cancer genes in detected deletion regions. **Table S3.** Comparison of results of Frankel to our results. **Table S4.** Comparison of results of Paulson to our results. **Table S5.** Comparison of results of Beroukhim to our results. **Table S6.** Comparison of results of Zack to our results. **Table S7.** Ploidy information of the 15 EA samples. (XLSX 57.4 kb)
